# Matrix Structure Evolution and Nanoreinforcement Distribution in Mechanically Milled and Spark Plasma Sintered Al-SiC Nanocomposites

**DOI:** 10.3390/ma7096748

**Published:** 2014-09-19

**Authors:** Nouari Saheb, Ismaila Kayode Aliyu, Syed Fida Hassan, Nasser Al-Aqeeli

**Affiliations:** Department of Mechanical Engineering, Center of Research Excellence in Nanotechnology, King Fahd University of Petroleum and Minerals, Dhahran 31261, Saudi Arabia; E-Mails: aismaila@kfupm.edu.sa (I.K.A.); sfhassan@kfupm.edu.sa (S.F.H.); naqeeli@kfupm.edu.sa (N.A.-A.)

**Keywords:** nanoreinforcement, distribution, matrix, crystallite size, strain, mechanical milling, spark plasma sintering, nanopowders, nanocomposites

## Abstract

Development of homogenous metal matrix nanocomposites with uniform distribution of nanoreinforcement, preserved matrix nanostructure features, and improved properties, was possible by means of innovative processing techniques. In this work, Al-SiC nanocomposites were synthesized by mechanical milling and consolidated through spark plasma sintering. Field Emission Scanning Electron Microscope (FE-SEM) with Energy Dispersive X-ray Spectroscopy (EDS) facility was used for the characterization of the extent of SiC particles’ distribution in the mechanically milled powders and spark plasma sintered samples. The change of the matrix crystallite size and lattice strain during milling and sintering was followed through X-ray diffraction (XRD). The density and hardness of the developed materials were evaluated as function of SiC content at fixed sintering conditions using a densimeter and a digital microhardness tester, respectively. It was found that milling for 24 h led to uniform distribution of SiC nanoreinforcement, reduced particle size and crystallite size of the aluminum matrix, and increased lattice strain. The presence and amount of SiC reinforcement enhanced the milling effect. The uniform distribution of SiC achieved by mechanical milling was maintained in sintered samples. Sintering led to the increase in the crystallite size of the aluminum matrix; however, it remained less than 100 nm in the composite containing 10 wt.% SiC. Density and hardness of sintered nanocomposites were reported and compared with those published in the literature.

## 1. Introduction

The quest to enhance the strength and stiffness of metals and alloys had led to the development of metal matrix composites (MMCs) [1,2] wherein the matrix is reinforced with particles, whiskers, or fibers. In particle reinforced MMCs, the embedding of hard and stiff ceramic particles in ductile and tough matrices improves not only the mechanical properties but also the physical properties of the composites. MMCs are widely used in automobile and aerospace industries because of their high specific modulus, strength-to weigh-ratio, fatigue strength, temperature stability and wear resistance [2,3,4]. The successful production of nano reinforcements *i.e*., particles with sizes less than 100 nm [5]; and the ability to decrease the crystallite size of the matrix to nano dimension paved the way for the development of metal matrix nanocomposites (MMNCs) which have better properties compared to MMCs. Research on MMNCs [6,7,8] has been intensified to overcome some of the challenges associated with their processing and achieve the desired properties. Amongst these challenges are the uniform distribution/dispersion of the nano-size reinforcement [8] and growth of the matrix crystallite size [7]. In MMNCs, the reinforcement is usually dispersed in the matrix either through melt or powder technologies [9]. In the former, the poor wettability of the particles by the melt [10,11] and formation of secondary brittle phases are the dominant challenges. In the later, uniform dispersion of the nanoreinforcement [8] and grain growth during sintering are the major drawbacks.

On the one hand, the use of powder metallurgy processing techniques such as ball milling, mechanical milling/alloying [12] resulted in the synthesis of nanocomposite powders [13] with uniform distribution of the nano-size reinforcement. Moreover, it enabled the production of nanostructured matrices such as copper [14,15,16], nickel [15], tungsten [17], cobalt [18], magnesium [19], Al-Mg [20], and aluminum [21,22]. On the other hand, the use of novel consolidation techniques such as spark plasma sintering (SPS) [7,23], also known as field assisted sintering (FAST), permitted sintering nanocomposites to full density with preserved nanostructure features of the matrix because of the high heating rates, short sintering cycles, and low sintering temperatures associated with the process [24,25,26,27]. In addition to being a single step process, the use of a binder is not required in the SPS process.

The SPS was used to prepare fully dense and high strength pure aluminum [28,29,30,31,32,33,34,35]. The high strength was attributed to both grain boundary and oxide dispersion strengthening [28,29,30]. The pinning effect, rapid heating cycle, and applied pressure were also found to play an important role in preventing particle growth [31]. The behavior of oxide film between the powder particles was reported to influence the properties of spark plasma sintered aluminum [32]. Aluminum alloys [36,37] have low weight and good properties; as a result, they are used in many engineering applications including automotive and aerospace. Their properties can be improved through the addition of SiC either micron-sized [38,39,40] or nano-sized [9,41,42,43]. Al-SiC nanocomposite powders were mainly synthesized using ball milling technique [9,38,44,45,46,47,48,49] and consolidated through different techniques such as double pressing/sintering process [50], hot extrusion [51], and spark plasma sintering [9,38,42,43,48].

In a very recent work [8], the authors reviewed nanoreinforcement dispersion in inorganic nanocomposites and found that the extent of nanoreinforcement dispersion in these nanocomposites is one of the sources of considerable discrepancy between their theoretically predicted and experimentally observed properties. The authors concluded that more research work is needed to develop homogenous nanocomposites, by means of innovative processing techniques, with uniform distribution of nanoreinforcements and improved properties. Despite the importance of Al-SiC nanocomposites, only few published works were dedicated to their synthesis using mechanical milling and consolidation through spark plasma sintering. Furthermore, the matrix structure evolution and nanoreinforcement distribution in both mechanically milled and spark plasma sintered Al-SiC nanocomposites were not fully investigated. The first objective of this work is to synthesize homogenous Al-SiC nanocomposite powders with uniform distribution of nano-sized SiC particles and nanostructured aluminum matrix through mechanical milling. The second objective is to consolidate the milled nanopowders through spark plasma sintering and explore the possibility to maintain the uniform distribution of the reinforcement and the nanostructure features of the matrix in the sintered nanocomposites. The influence of SiC content on the density and properties of the developed composites will be investigated.

## 2. Materials and Experimental Procedures

### 2.1. Materials

Aluminum powder of 99.88% purity, supplied by (supplied by the Aluminum Powder Co. Ltd., West Midlands, UK), and SiC_β_ (45–55 nm) of 97.5% purity, supplied by Nanostructured and Amorphous Materials (Houston, TX, USA) were used in this investigation. The chemical composition and particle size distribution of the aluminum powder are presented in Table 1 and Table 2, respectively.

**Table 1:** Chemical composition of pure aluminum powder.

Elements	Al	Fe	Si	Ti	Ga	Ni	Cu, Mn, Pb, Zr, Zn, Cr
wt. %	99.88	0.074	0.024	0.006	0.006	0.005	0.001 each

**Table 2:** Particle size distribution of aluminum powder.

Size (µm)	%
63	0
53	1
45	11
38	11.4
<38	76.6

### 2.2. Experimental Procedures

Al-SiC nanocomposite powders containing 1, 5 and 10 wt.% SiC were prepared through mechanical milling; one of the most important techniques used to synthesis nanocomposite powders at the solid state [13]. It involves cold welding, fracturing and rewelding of powder particles. The milling experiments were carried out using a planetary ball mill (Fritsch Pulverisette, P5, Idar-Oberstein, Germany), at room temperature, in argon atmosphere to prevent the oxidation of the powders. A ball to powder weight ratio (BPR) of 10:1 and a speed of 200 rpm were used. Stearic acid was used as process control agent (PCA) to minimize sticking of the powder to milling tools. The powder mixture was charged into cylindrical stainless steel vials (250 mL in volume) together with stainless steel balls (10 mm in diameter) and milled for different milling times; at each time, a small amount of the powder was taken out of the vial for characterization and analysis. A Tescan Lyra-3 Field Emission Scanning Electron Microscope (FE-SEM) with Energy Dispersive X-ray Spectroscopy (EDS) facility was used for the characterization of the mechanically milled powders. X-ray mapping was performed using constant number of frames for elemental mapping to characterize the extent of particles’ distribution in the milled nanocomposite powders as well as sintered samples. A high resolution X-ray diffractometer (Bruker D8, Madison, WI, USA, with a wavelength λ = 0.15405 nm) was used to record the X-ray diffraction (XRD) patterns of the milled powders to analyze the change of crystallite size of the Al matrix in milled and sintered samples. Milling involves the reduction of crystallite size which leads to X-ray diffraction peak broadening, and induces lattice plane distortion (strain). Crystallite size and lattice strain of the Al matrix during milling were evaluated using the following equation [52].

*B_r_*cosθ = k λ/L + ηsinθ (1)

where *B_r_* is the full width at half-maximum (FWHM) of the diffraction peak after instrument correction, *k* is constant (with a value of 0.9); λ is wavelength of the X-ray radiation (λ = 0.15405 nm); *L* and *η* are crystallite size and lattice strain, respectively; and θ is the Bragg angle. 

The B_r_ is related to the measured width of the peak (*B_obs_*) and peak broadening caused by factors except the crystallite size effect (*B_i_*), frequently called instrumental broadening factor and calculated using a fully annealed sample, through the following formula:

(2) $$B_{r}^{2}=B_{obs}^{2}-B_{i}^{2}$$

The above Equations (1) and (2) are frequently used to evaluate crystallite size change and strain accumulation [44,46,47] in mechanically milled materials.

The prepared nanocomposite powders were consolidated through spark plasma sintering using a fully automated equipment (FCT system, Rauenstein, Germany), model HP D 5. The powder was directly charged into a 20 mm graphite die through which the current was passed. The sintering temperature was measured using a thermocouple inserted in the graphite die through a drilled hole. A graphite sheet was used to minimize friction between the die walls and the powder as well as to ease the ejection of the sample after the sintering has been completed. Samples were sintered at 600 °C for 10 min under an applied pressure of 50 MPa using a heating rate of 200 °C/min.

The density of the sintered samples was measured using an Alfa Mirage electronic densimeter, model MD-300 s (accuracy of 0.001 g/cm^3^) and quantified according to Archimedes principle. Vickers microhardness of the spark plasma sintered samples was measured using a digital microhardness tester (Buehler, Lake Bluff, IL, USA). All measurements were conducted by applying the same conditions: a load of 100 gf, a time of 12 s. The data reported were the average of 10 values.

## 3. Results and Discussion

**Figure 1:**
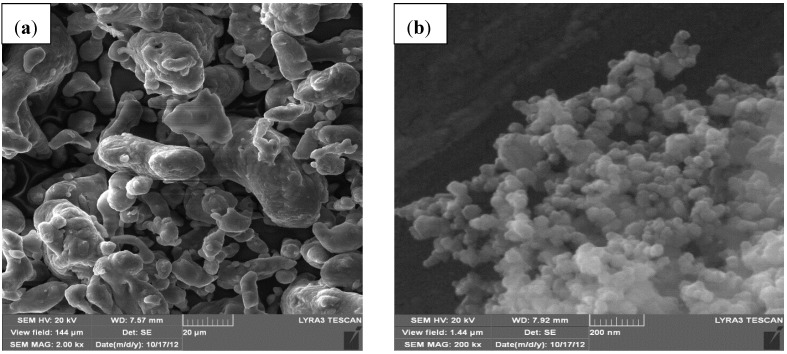
FE-SEM micrographs of as received powders (**a**) Al and (**b**) SiC.

Figure 1 shows FE-SEM micrographs of the as-received Al powder and SiC nanopowder. The aluminum powder consists of particles with various shapes including spherical, elongated and irregular as shown in Figure 1a. Analysis of its particle size distribution showed a maximum particle size of 53 μm; and 76.6% of particles have a particle size less than 38 μm. The SiC nanopowder, Figure 1b, consists of particles with irregular shapes but close to spherical with a mean of 50 nm. The SiC nanoparticles were more agglomerated than the Al particles because of their small size.

**Figure 2:**
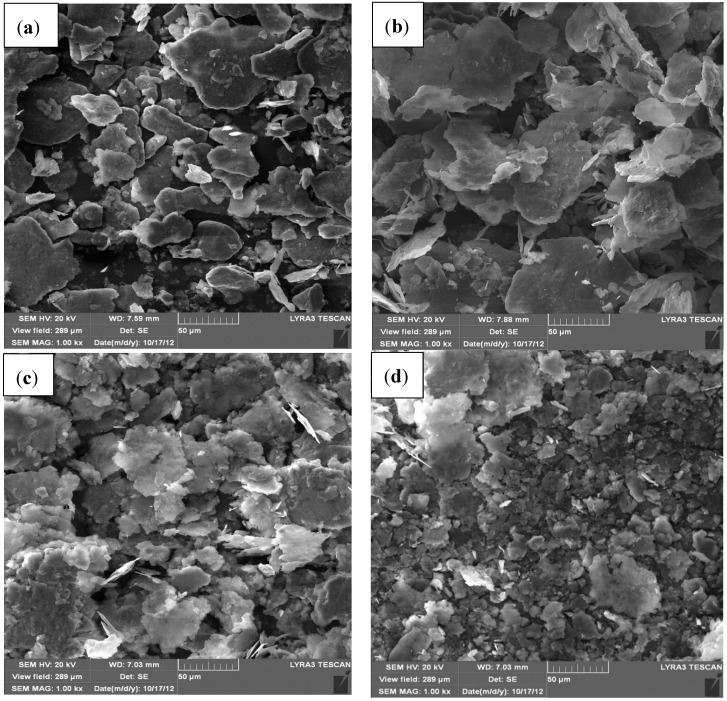
FE-SEM micrographs of Al-5 wt.% SiC nanocomposite powder milled for (**a**) 2 h; (**b**) 9 h; (**c**) 20 h; (**d**) 24 h.

The evolution of particles’ morphology of Al-5 wt.% SiC powder milled for different times is presented in Figure 2. Overall, mechanical milling of the nanocomposite powder for 24 h reduced the particle size of the aluminum powder.

**Figure 3:**
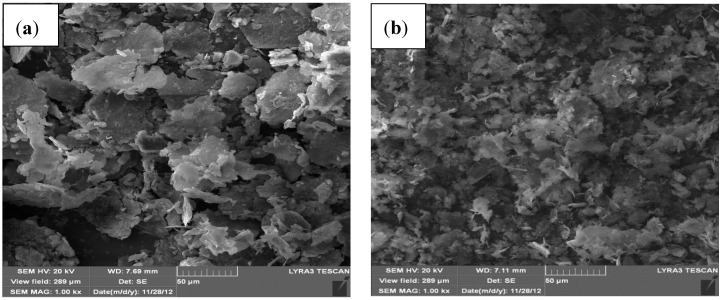
FE-SEM micrographs of (**a**) Al-1 wt.% SiC and (**b**) Al-10 wt.% SiC nanocomposite powders milled for 24 h.

A similar behavior was observed in nanocomposite powders containing 1 and 10 wt.% of SiC where a milling time of 24 h deceased the size of Al particles as shown in Figure 3a,b, respectively, compared to the un-milled powder presented in Figure 1a.

Comparing Figure 3a,b and Figure 2d, showing morphology and size of Al particles in nanocomposites milled for 24 h and containing 1, 5, and 10 wt.% SiC, respectively; one can conclude that the presence of SiC nanoparticles enhanced the milling effect. This is in agreement with the fact that the increase in SiC content increases the rate of milling and decreases the time to reach a steady state regime [50,53] where fracturing and rewelding of particles are balanced. Khadem and co-workers [49] reported that the addition of 5 vol.% of SiC nanoparticles to pure aluminum improved milling and led to faster work hardening and fracture of the aluminum matrix. The higher grinding effect was also observed at higher content of SiC nanoparticles when aluminum alloy nanocomposites reinforced with 5, 12 and 20 wt.% nano-SiC particles (20–40 nm) were ball milled at speed of 200 rpm for up to 20 h using a BPR of 10:1 [48].

**Figure 4:**
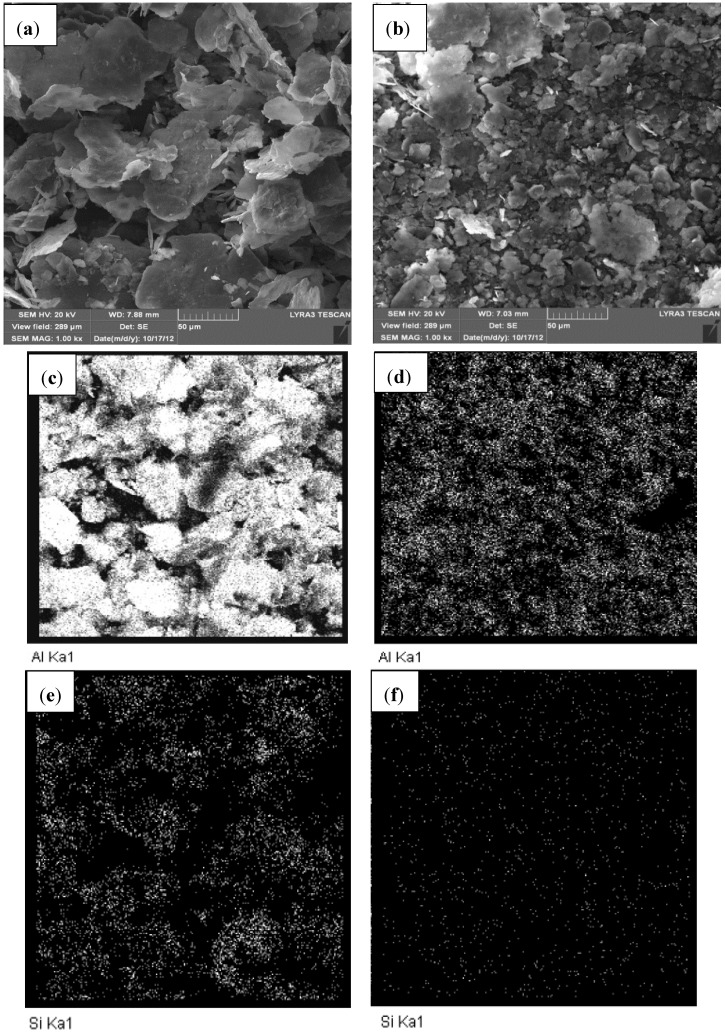
(**a**) FE-SEM micrographs of Al-5 wt.% SiC nanocomposite powder milled for (**a**) 9 h; (**b**) 24 h; mapping of Al after (**c**) 9 h; (**d**) 24 h; and mapping of Si after (**e**) 9 h; (**f**) 24 h.

A typical X-ray mapping of Al-5 wt.%SiC nanocomposite powder milled for 9 and 24 h is presented in Figure 4. Figure 4a,b shows FE-SEM micrographs of the nanocomposite milled for 9 and 24 h, respectively. It can be clearly seen that the increase of milling time from 9 to 24 h reduced the particle size and promoted the formation of more equiaxed particles as discussed above. In addition, it can be noticed that after milling for 9 h, Al particles remained relatively large and SiC nanoparticles are still agglomerated as confirmed by mapping of Al and Si elements shown on Figure 4c,e, respectively. The increase of milling time to 24 h decreased the size of Al particles, reduced the agglomeration of SiC particles and improved their dispersion; and a homogenous nanocomposite powder was obtained. The uniform dispersion was confirmed through mapping of Al and Si elements as shown on Figure 4d,f, respectively. The same trend was observed in mechanically milled Al-1 wt.% SiC and Al-10 wt.% SiC nanocomposites where a milling time of 24 h yielded homogenous nanocomposite powders with uniform distribution of SiC particles. The milling time was longer compared to 30 min reported by Yadav [45] to reach a uniform dispersion of SiC particles in milled Al-SiC nanocomposites containing 5, 10 and 20 wt% SiC. The author used acetone as PCA and polyacrylic as dispersive agent, a BPR of 5:1, and a higher speed of milling of 500 rpm. In other work [44], the authors used a speed of 320 rpm and BPR of 20:1 and 10:1; and reported a milling time of 10 h to reach a uniform distribution SiC in ball milled Al-SiC nanocomposites containing 2.5, 7.5 and 12.5 vol. % SiC. This clearly shows that the time to achieve uniform distribution of the reinforcement is function of milling conditions such as the BPR and milling speed. However, high BPR ratio and milling speed may lead to contamination of the powder from milling tools.

**Figure 5:**
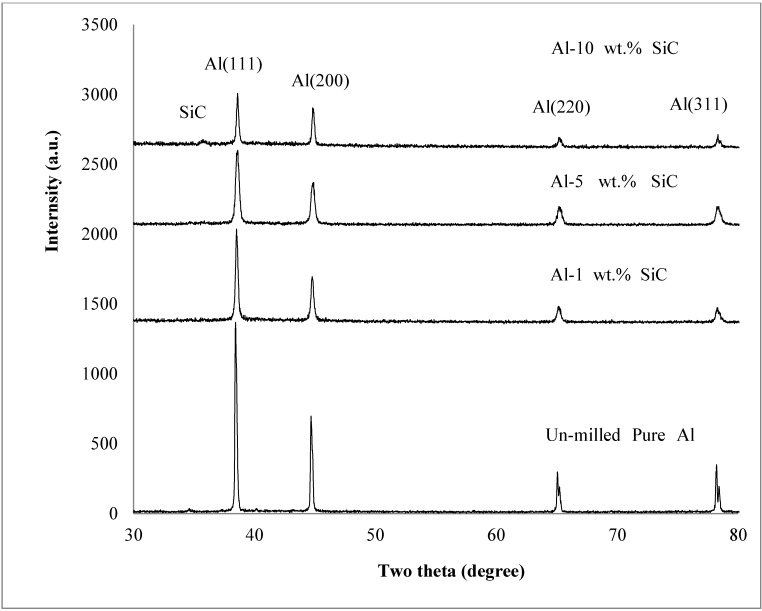
XRD spectra of the as-received aluminum and Al-SiC nanocomposite powders mechanically milled for 24 h containing.

X-ray diffraction spectra of the as-received un-milled Al powder and Al-SiC nanocomposite powders milled for 24 h are presented in Figure 5. All spectra were referred to the same scale for comparison. The un-milled pure aluminum has face centered cubic crystal structure, other elements present in the form of impurities as presented in Table 1, are in solid solution in α-Al. Milling of the composite powders containing 1, 5, and 10 wt.% of SiC nanoparticles led to the decrease of the intensity and broadening of the peaks of the α-Al phase. This is attributed to the decrease of the crystallite size and increase of lattice strain of the Al matrix associated with milling. Usually, in mechanically milled powders, crystallite size reduction takes place in three stages. The first stage is characterized by the formation of shear bands with high density of dislocations. In the second stage, annihilation and recombination of these dislocations give rise to small angle grain boundaries separating the individual grains. In the last stage, the orientation of the single crystalline grains become random with respect to their neighboring grains [54]. It is worth mentioning here that, “in XRD analysis, when the size of a crystal is used, it usually refers to the size of crystallites concerning a factor, which makes a diffraction peak broad” [52].

The XRD spectra of the milled Al-SiC nanocomposite powders did not reveal the formation of secondary brittle phases such as Al_4_C_3_. This is may be because, from one side, the composites are prepared through powder technology and not melt technology; and from the other side the milling decreases the intensity of peaks [55]. Therefore, the amount of compounds, if present, may not be enough to be clearly revealed on the XRD spectra [56]. The absence of Al_4_C_3_ in mechanically milled Al-SiC nanocomposites had been confirmed by other researchers even at high milling speeds of 320 rpm [44] and 500 rpm [45]. Furthermore, Figure 5 revealed the presence of only one peak of the SiC phase in the XRD spectrum of Al-10 wt.% SiC nanocomposite powder milled for 24 h. The absence of SiC peaks could be attributed, from one side, to their very small crystallite size, which makes peaks very broad; and from the other side, to the low volume fraction of the SiC phase. In addition, peak broadening can result from plastic deformation and the increase of the nonuniform strain of the milled samples [48,52,57].

**Figure 6:**
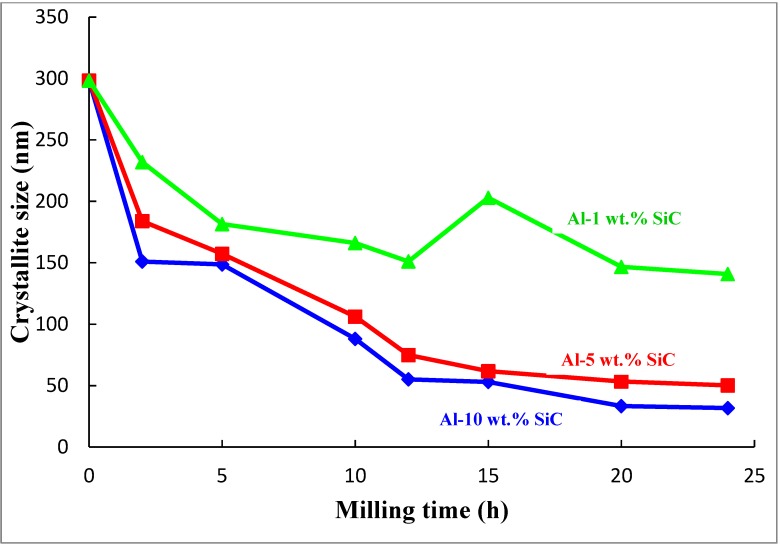
Change of aluminum matrix crystallite size as function of milling time.

The change of the matrix crystallite size as a function of milling time is presented in Figure 6.

In addition to reducing the particle size as discussed above, milling decreased the crystallite size of the α-aluminum. It can be clearly seen, in Figure 6, that milling of the powders for 24 h decreased the crystallite size of the α-aluminum phase from around 300 to 140, 50, and 32 nm, in nanocomposites containing 1, 5, and 10 wt.% SiC, respectively. The crystallite size of the aluminum matrix is within the range reported by other researchers for the nanostructured aluminum phase obtained through mechanical milling either the monolithic metal [21,22] or Al-SiC nanocomposites [42,43,44]. Dry milling of pure aluminum powder was found to decrease its crystallite size to 38 nm in 8 h [21] and 75 nm in 20 h [22]. For Al-SiC nanocomposites containing 12.5 and 2.5 vol.%, and milled for 10 h, the authors reported matrix crystallite sizes of 175.6 and 97.9 nm, respectively [44]. In Al5083-10 wt.% SiC nanocomposites, ball milled for 15 h at a speed of 400 rpm using a BPR of 20:1; a crystallite size of 25 nm was obtained [42,43].

**Figure 7:**
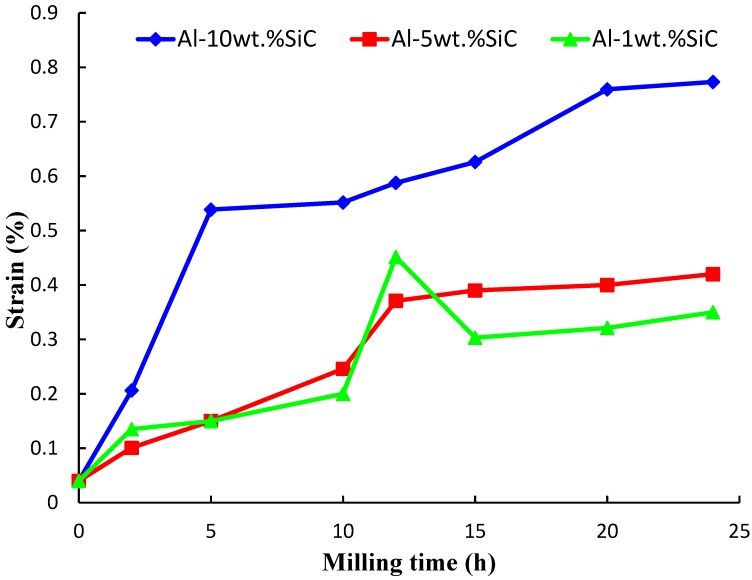
Change of strain in the aluminum matrix as function of milling time.

Strain in the aluminum matrix as a function of milling time is presented in Figure 7. Milling of the powders for 24 h increased the lattice strain of the α-aluminum phase from 0.04 to 0.35, 0.42, and 0.77%, in nanocomposites containing 1, 5, and 10 wt.% SiC, respectively. This is in agreement with the fact that during mechanical grinding processes such as mechanical milling nonuniform strain is accumulated because of the plastic deformation of the structure [48,57]. Lattice strain of the pure aluminum matrix in the nanocomposite containing 10 wt.% SiC *i.e*., 0.77 was slightly higher than the lattice strain of 0.43 reported by Bathula and co-workers [43] for Al5083 matrix reinforced with 10 wt.%SiC and milled for 15 h at a speed of 400 rpm using a BPR of 20:1.

It is evident from Figure 6 and Figure 7 that the major reduction in crystallite size and increase in lattice strain, respectively, of the α-aluminum phase occurred during the first 12 h of milling after which the change was not significant. This is because after a period of milling, the rate of grain refinement and particle fracturing becomes equal to the rate of grain growth and recovery. Furthermore, the smaller grains are already saturated with defects and dislocation pile-ups and, hence, the lattice structure cannot continue to develop the same way as is known for coarse-grained metals [22]. As soon as the nanostructure is formed, further decrease in crystallite size become more difficult because of the large stress required to continue its deformation. Therefore, the creation and movement of dislocations under these circumstances will be more difficult. Crystallite size reduction during mechanical milling may also be limited because of recovery especially for metals with a low melting point [19].

Analysis of the evolution of the α-aluminum crystallite size as a function of milling time showed that a milling time of 12 h is suitable time to reach a nanostructured matrix. However, uniform distribution of SiC nanoparticles was achieved at 24 h of milling. Therefore, the Al-SiC nanocomposite powders milled for 24 h were spark plasma sintered at fixed sintering conditions to investigate their densification and characterize their microstructure features.

**Figure 8:**
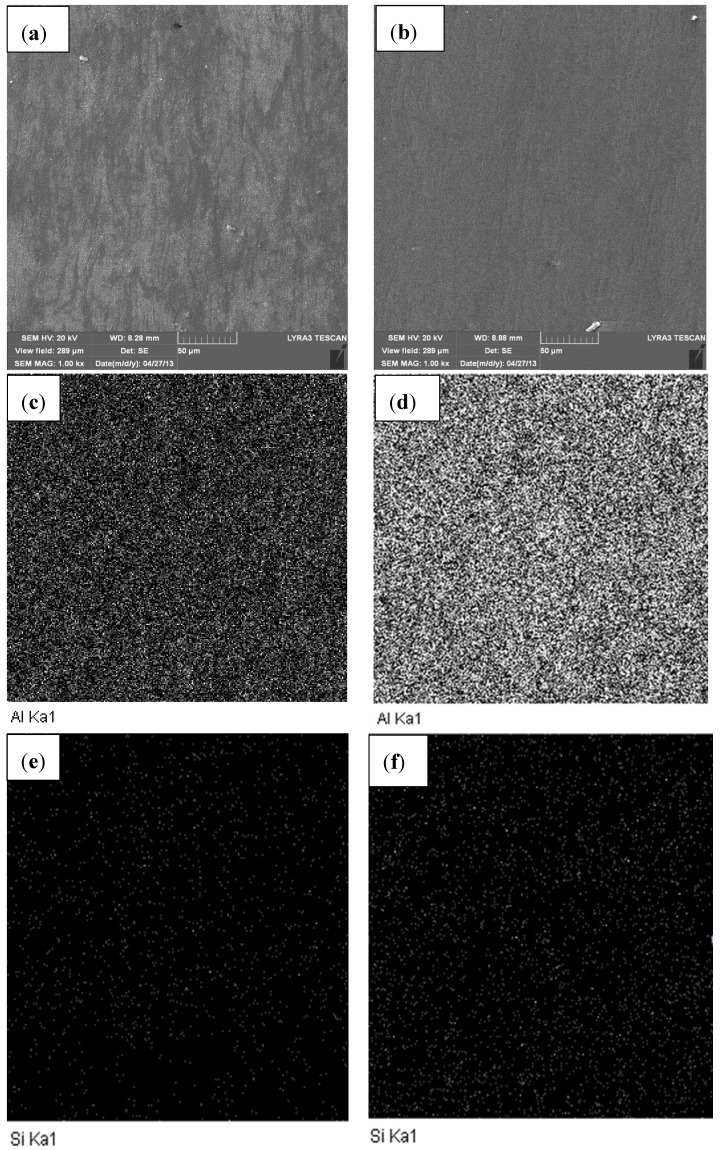
(**a**) FE-SEM micrographs of sintered (**a**) Al-5 wt.% SiC; (**b**) Al-10 wt.% SiC nanocomposites; mapping of Al in (**c**) Al-5 wt.% SiC (**d**) Al-10 wt.% SiC; mapping of Si in (**e**) Al-5 wt.% SiC (**f**) Al-10 wt.% SiC.

Figure 8a,b show FESEM micrographs of Al-5 wt.% SiC and Al-10 wt.% SiC nanocomposites sintered at 600 °C for 10 min under an applied pressure of 50 MPa using a heating rate of 200 °C/min. Mapping of Al in composites containing 5 and 10 wt.% of SiC is presented in Figure 8c,d, respectively. Mapping of Si in the sintered samples confirmed that the uniform distribution of SiC achieved by mechanical milling was maintained in sintered samples as clearly seen in Figure 8e,f for SiC content of 5 and 10 wt.%, respectively.

**Figure 9:**
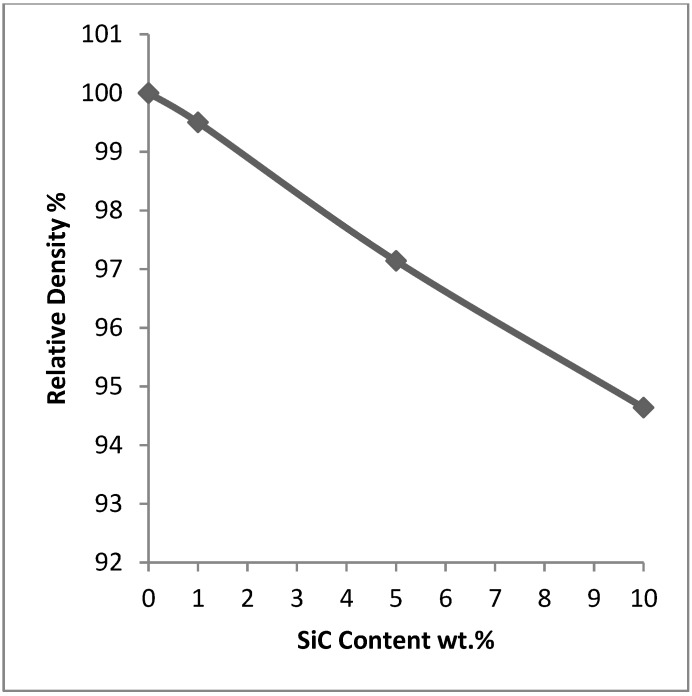
The relative density of samples sintered at 600 °C for 10 min under an applied pressure of 50 MPa using a heating rate of 200 °C/min.

The relative density of samples sintered at 600 °C for 10 min under an applied pressure of 50 MPa using a heating rate of 200 °C/min is presented in Figure 9. Pure aluminum was fully densified *i.e*., the relative density reached 100%. The nanocomposites displayed less densification compared with the monolithic pure metal. The addition of 1 wt.% SiC decreased the relative density to 99.59%. The increase of SiC to 5 wt.% decreased the relative density to 97.14%. Further increase of SiC content to 10 wt.% decreased the relative density to 94.64%. The decrease of the relative density of the nanocomposites with the increase of SiC content is in agreement with the fact that nanostructured samples are likely to be more porous [9]. It is believed that compaction of powder containing grains of nanometer dimensions, especially when ceramic or carbide nanoparticles are added is more difficult than the compaction of micron-sized powder of the same metal or alloy [50,53]. This is because plasticity in nanocrystalline samples requires order of magnitude larger stresses and the spring back is more pronounced.

Sintering increased the crystallite size of pure aluminum from 298 to 366 nm. As for the nanocomposites, the crystallite size increased from 140 to 298 nm for sample containing 1 wt.% SiC, 50 to 144 for sample containing 5 wt.% SiC, and 32 to 66 nm for sample containing 10 wt.% SiC. This shows that the higher the SiC content, the lower the crystallite size of the aluminum matrix. Therefore, it can be concluded that the presence and amount of SiC contributed to the inhibition of grain growth. The crystallite size of the aluminum matrix is within the range reported by other researchers for pure aluminum and Al-based nanocomposites reinforced with SiC or other reinforcements and processed through spark plasma sintering or other methods as summarized in Table 3.

**Table 3:** Crystallite size of the aluminum matrix in the nanocomposites before and after sintering compared to published data in the literature.

Composite	Sintering conditions	*L* before sintering (nm)	*L* after sintering (nm)	Ref.
Al-10 wt.% SiC	SPS, 600 °C, 50 MPa, 10 min	32	66	This work
Al-5 wt.% SiC	SPS, 600 °C, 50 Mpa, 10 min	50	144	This work
Al-1 wt.% SiC	SPS, 600 °C, 50 Mpa, 10 min	140	298	This work
Pure Al	SPS, 600 °C, 50 Mpa, 10 min	298	366	This work
Al-SiC	SPS, 450 °C, 200 MPa, 5 min	-	100	[9]
Al-6 wt.% SiC	CIP, 640 °C, 700 MPa, 1 h	69	350	[58]
Al-1.2 wt.% SiC	CIP, 640 °C, 700 MPa, 1 h	63	150	[58]
Al-10 SiC-CNT	Hot pressing for 1.5 h, 550 °C	32	43	[59]
Al-5 SiC-CNT	Hot pressing for 1.5 h, 550 °C	34	45	[59]
Pure Al	Hot pressing for 1.5 h, 550 °C	141	183	[59]
Al-5356/B4C	SPS, 500 °C, 50 MPa, 5 min	36	92	[60]
Al5083-10 wt.% SiC	SPS, 500 °C, 50 MPa, 3 min	25	30	[43]

**Figure 10:**
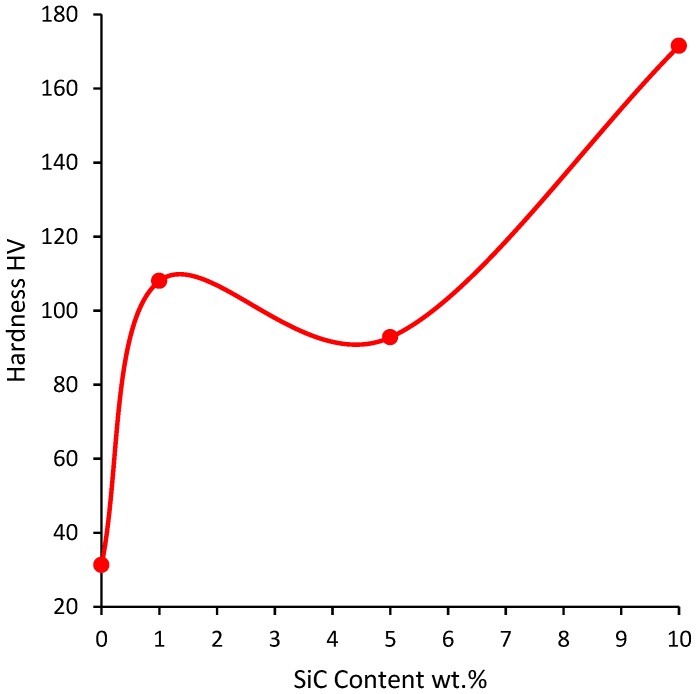
Hardness of samples sintered at 600 °C for 10 min under an applied pressure of 50 MPa using a heating rate of 200 °C/min.

The hardness of samples sintered at 600 °C for 10 min under an applied pressure of 50 MPa using a heating rate of 200 °C/min is presented in Figure 10. The monolithic pure aluminum had a hardness of 31.3 HV. Although it was fully densified it displayed low hardness, this is due to the increase of its crystalline size at the relatively high sintering temperature of 600 °C. The addition of 1 wt.% SiC increased hardness to 108 HV. The increase of SiC to 5 wt.% decreased the hardness to 92.8 HV. However, further increase of SiC content to 10 wt.% increased hardness to 171.53 HV. The higher hardness displayed by the nanocomposite reinforced with 10 wt.% SiC is due to the uniform distribution of the reinforcement and the small crystallite size of the matrix induced by milling and maintained during sintering.

The hardness of the Al-10 wt.% SiC nanocomposite was higher than the hardness of other nanocomposites reinforced either with CNTs or SiC. These include Al2124 + 1 wt.% CNTs [61], Al6061 + 1 wt.% CNTs [62], Al-7Si-0.3Mg+0.5CNTs [51], Al-12Si-0.3Mg + 0.5CNTs [63], and Al-7Si-0.3Mg + SiC [48]. However, it was lower than the hardness of Al5083-10 wt.% SiC [43]. A summary of Vickers hardness values of selected spark plasma sintered aluminum based nanocomposites is presented in Table 4. This data shows that the hardness of spark plasma sintered Al-based nanocomposites depends on the matrix type, whether pure aluminum or alloy, as well as sintering parameters.

In summary, milling of the Al-SiC nanocomposites led to uniform distribution of the SiC reinforcement and reduced the crystallite size of the aluminum matrix to less than 140 nm. The sintered nanocomposites maintained the uniform distribution of SiC particles and displayed good densification. SiC inhibited grain growth of the matrix and increased the hardness of the composites. The influence of sintering parameters particularly heating rate, compaction pressure, sintering temperature and time, on the structure and mechanical properties of spark plasma sintered Al-SiC nanocomposites will be the subject of future studies.

**Table 4:** Vickers Hardness of SPS sintered nanocomposites compared to published data in the literature.

Composite	K (°C/min)	P (MPa)	T (°C)	Time (min)	HV	Ref.
Pure Al	200	50	600	10	31.3	This work
Al-1 wt.% SiC	200	50	600	10	108	This work
Al-5 wt.% SiC	200	50	600	10	92.8	This work
Al-10 wt.% SiC	200	50	600	10	171.53	This work
Al5083	300	50	500	3	148	[43]
Al5083-10 wt.% SiC	300	50	500	3	250	[43]
Al2124	100	35	500	20	110.24	[61]
Al2124 + 1 wt.% CNTs	100	35	500	20	118.19	[61]
Al6061	100	35	450	20	66	[62]
Al6061 + 1 wt.% CNTs	100	35	450	20	71	[62]
Al-7Si-0.3 Mg	100	35	500	20	63	[63]
Al-7Si-0.3 Mg + 0.5 wt.% CNTs	100	35	500	20	68	[63]
Al-12Si-0.3 Mg	100	35	500	20	68	[63]
Al-12Si-0.3 Mg + 0.5 wt.% CNTs	100	35	500	20	83	[63]
Al-7Si-0.3 Mg	100	35	500	20	63	[48]
Al-7Si-0.3 Mg + 5 wt.% SiC	100	35	500	20	71	[48]
Al-7Si-0.3 Mg + 12 wt.% SiC	100	35	500	20	75	[48]
Al-7Si-0.3 Mg + 20 wt.% SiC	100	35	500	20	69	[48]

## 4. Conclusions

Al-SiC nanocomposites containing 1, 5, and 10 wt.% SiC were successfully synthesized by mechanical milling and consolidated through spark plasma sintering. Milling of the powders for 24 h led to uniform distribution of SiC nanoparticles and decreased the crystallite size of the α-aluminum phase from around 300 to 140, 50, and 32 nm, in nanocomposites containing 1, 5, and 10 wt.% SiC, respectively. This yielded a nanostructured aluminum matrix. The uniform distribution of SiC achieved by mechanical milling was maintained in sintered samples. Sintering led to the increase in the crystallite size of the aluminum matrix, however, it remained less than 100 nm in the composite containing 10 wt.% SiC. The nanocomposite reinforced with 10 wt.% SiC had a relatively high Vickers hardness value of 171.53 HV.
